# Assessing work-related fatigue and burden among Egyptian oncology nurses: a cross-sectional study

**DOI:** 10.1186/s12889-025-23374-z

**Published:** 2025-06-25

**Authors:** Ghada O. El-Khawaga, Abdel-Hady El-Gilany, Heba Ali Hamed Mohamed

**Affiliations:** 1 Public Health and Community Medicine Department, Faculty of Medicine, Mansoura University, Mansoura, Egypt; 2 Faculty of Medicine, Mansoura National University, Mansoura, Egypt; 3 Alghad Colleges for Applied Medical Sciences, Al Madinah Al Munawwara, Medina, Saudi Arabia; 4 Community Health Nursing department, Faculty of Nursing, Mansoura University, Mansoura, Egypt

**Keywords:** Work-related fatigue, Work burden, Oncology, Egyptian nurses, Occupational health, Nursing stress

## Abstract

**Background:**

Despite increasing attention to nurse fatigue globally, little is known about its prevalence and contributing factors among Egyptian oncology nurses. Work-related fatigue and burden not only affect nurses’ well-being but also compromise patient care quality and healthcare system efficiency. This study aimed to assess the prevalence of work-related fatigue and burden among oncology nurses in Egypt and to identify their associated factors.

**Methods:**

An observational cross-sectional study was conducted at the Mansoura Oncology Center in Egypt. A convenience sampling method was used to recruit 273 nurses during their work shifts. Data were collected through a structured face-to-face questionnaire, incorporating socio-demographic details and the Arabic version of the “Self-diagnosis Checklist for Assessment of Workers’ Accumulated Fatigue”. Data were analyzed using SPSS version 27, employing descriptive statistics, chi-square tests, and logistic regression to identify significant predictors.

**Results:**

The mean fatigue score among participants was 16.2 ± 7.5, with 24.5% of nurses experiencing grade IV fatigue. Regarding work burden, 68.1% of nurses reported grade D burden. Multivariate analysis revealed that being married, holding a bachelor’s degree or higher, and having less than 10 years of work experience were independent predictors of grade IV fatigue, with adjusted odds ratios (AOR) of 2.8, 2.2, and 2.4, respectively. Grade D burden was independently associated with being unmarried (AOR = 2.2) and reporting insufficient income (AOR = 2.8).

**Conclusions:**

The findings highlight a high prevalence of severe work-related fatigue and burden among oncology nurses in Egypt. Addressing these challenges through institutional policies, adequate staffing, financial support, and targeted mental health interventions is essential. Further research should explore causal relationships and implement interventions to mitigate fatigue and burden in oncology nursing practice.

## Introduction

Oncology nursing is a specialized field that demands high levels of clinical expertise, emotional resilience, and sustained compassion in caring for patients with cancer [1]. Globally, oncology nurses are integral to cancer care, providing direct patient care, symptom management, psychological support, and end-of-life care, often under emotionally and physically challenging conditions [2]. According to the World Health Organization, nurses, including those specializing in oncology, constitute the largest segment of the health workforce and play an indispensable role in achieving optimal health outcomes [3]. The American Nurses Association emphasizes that nurses contribute significantly beyond bedside care, engaging in patient education, research, and advocacy to ensure holistic care for individuals with complex health conditions such as cancer [4].

However, oncology nurses are particularly susceptible to work-related fatigue due to the demanding nature of their work. They frequently manage critically ill patients, navigate emotionally taxing patient and family interactions, and operate within high-stress environments marked by staffing shortages and administrative pressures [5]. These challenges are compounded by extended shifts, insufficient rest periods, and emotional exhaustion, contributing significantly to both physical and mental fatigue [6].

Fatigue itself is a multifaceted phenomenon lacking a single universally accepted definition [7]. Generally, it encompasses physical exhaustion, mental tiredness, and emotional depletion resulting from prolonged stress or overwork [8]. Fatigue is increasingly recognized as a significant occupational health issue in healthcare settings, yet its seriousness is often trivialized or overlooked [9]. Oncology nurses, in particular, endure cumulative fatigue from irregular work schedules, night shifts, and the emotional labor of dealing with patients’ suffering and death [10].

In oncology settings, evidence shows that work-related fatigue among nurses leads to decreased quality of care, increased clinical errors, and higher turnover rates, directly impacting patient safety and healthcare costs [11]. A systematic review by Gomez-Urquiza et al. (2017) found that burnout rates among oncology nurses are among the highest across nursing specialties globally [12].

In Egypt, these global challenges are further magnified by specific systemic issues. The Egyptian healthcare system has faced persistent problems such as chronic underfunding, high patient-to-nurse ratios, limited resources, and substantial workforce migration [13]. Nurses in Egypt, including those working in oncology, often work extended hours under stressful conditions with insufficient support, exacerbating the risk of fatigue and burnout [14]. According to the Central Agency for Public Mobilization and Statistics (CAPMAS), Egypt faces a shortage of trained nurses, with nurse-to-population ratios significantly below WHO recommendations [15]. Furthermore, a study by Mohammed et al. (2023) revealed that 36% of studied oncology nurses in Egypt reported high levels of total work stress, with fatigue cited as a primary consequence [16].

Despite extensive international research into nurse fatigue, there is a notable lack of focused studies on oncology nurses in Egypt and the broader Arab region. Existing research tends to generalize findings across different nursing specialties without isolating the unique occupational stressors encountered in oncology care [17, 18]. For instance, a study conducted in China found that nurses aged 30–40 with 5–10 years of experience reported the highest levels of cumulative fatigue [19]; however, no comparable large-scale studies have been performed in Egypt to validate whether similar patterns exist locally.

This knowledge gap limits the ability of healthcare policymakers to implement evidence-based interventions tailored to the Egyptian oncology nursing context. Moreover, filling this gap is essential not only for improving local practices but also for contributing valuable data to the global body of knowledge on oncology nursing fatigue in low- and middle-income countries, where healthcare system dynamics differ significantly from those in high-income nations [20]. Therefore, the aim of this study is to measure the prevalence of work-related fatigue and burden among Egyptian oncology nurses and to identify associated socio-demographic and occupational factors. Addressing these issues is critical to safeguarding nurse well-being, maintaining patient safety, and enhancing the sustainability of oncology care services.

## Methods

### Study design

A cross-sectional design was chosen for this study as it allows for the assessment of fatigue and work burden at a single point in time, making it well-suited to the study’s objective of determining the prevalence and factors associated with work-related fatigue and burden among nurses in the oncology setting. It was conducted from April 1st to September 30th, 2024.

### Study setting and population

The study was conducted at Mansoura Oncology Center, a specialized facility providing comprehensive cancer care, including outpatient clinics, inpatient wards, intensive care units (ICU), operating rooms, radiotherapy, chemotherapy, palliative care, and diagnostic imaging. The target population consisted of all nurses working in direct contact with patients in these various departments. The inclusion criteria were nurses who were apparently healthy, had at least one year of work experience, and were actively engaged in patient care during the study period.

### Sample size calculation

Sample size was calculated using OpenEpi [21]. Based on a prior study that reported 23.1% of nurses experiencing high levels of fatigue [19], the required sample size was determined to be 273. The calculation was performed with a significance level (α) set at 0.05, a precision of 5%, and a statistical power (1-β) set at 0.80.

### Sampling method

A convenience sampling method was used to recruit participants. Nurses were approached during their shifts, and their participation was voluntary. The total number of nurses working at the center was 486, and 294 nurses were approached for participation. Of these, 273 nurses agreed to participate, yielding a response rate of 92.9%. The high response rate was achieved through face-to-face interactions, which facilitated rapport-building and encouraged participation. Measures were taken to minimize potential biases related to convenience sampling, including ensuring that the sample represented different departments (outpatient clinics, inpatient wards, ICU, operating rooms) within the center.

### Data collection procedure

Data were collected through face-to-face structured questionnaire administration conducted at the workplace. The interviewer explained the study objectives, and participants were asked to complete the structured questionnaire during their shifts, ensuring minimal disruption to their work. The questionnaire was designed to be easy to complete, and the interviewer was available to clarify any questions the participants had.

The questionnaire consisted of two parts:

#### Part I

Socio-demographic characteristics of the nurses, including age, gender, marital status, qualifications, monthly salary, years of work experience, and department of work.

**Part II**: The Arabic version of the “Self-diagnosis Checklist for Assessment of Workers’ Accumulated Fatigue” scale, which measures work-related cumulative fatigue [22]. This scale includes 13 items assessing fatigue symptoms (e.g., feeling sleepy, tiredness, lack of concentration), rated on a three-point frequency scale (0 = rarely, 1 = sometimes, 2 = often). Fatigue severity is categorized into four grades: I (0–4), II (5–10), III (11–20), and IV (21–39). The second part of the questionnaire also included seven items measuring work burden, such as workload, irregular schedules, and physical and mental work stress. These items were rated on a two- or three-point scale (low = 0, medium = 1, high = 3), and work burden was categorized into four levels: A (0), B (1–2), C (3–5), and D (6–15). The questionnaire was piloted on a small sample of nurses to ensure clarity and ease of administration before the full data collection. The subscales of fatigue symptoms and work burden had a Cronbach’s alpha coefficient of 0.931 and 0.813, respectively, in this study, indicating good internal consistency [19].

### Statistical analysis

The data collected were coded and analyzed using SPSS (Version 27). Descriptive statistics, including frequencies, percentages, means, and standard deviations, were calculated to summarize the socio-demographic characteristics of the participants, as well as their levels of fatigue and work burden. The Chi-square test was applied to examine associations between socio-demographic variables and fatigue/burden categories. To identify the independent predictors of fatigue and work burden, both univariate and multivariate logistic regression analyses were performed. Variables that were significant in the univariate analysis were entered into a multivariate logistic regression model using forward Wald techniques. Adjusted odds ratios (AOR) and 95% confidence intervals (CI) were computed to evaluate the strength and significance of the associations between predictors and outcomes. The statistical methods included the Chi-square test for categorical variables to test significant associations and logistic regression for assessing the predictors of fatigue and burden. Both crude odds ratios (COR) and adjusted odds ratios (AOR) with 95% CI were used to quantify the relationships between the variables of interest. A p-value of ≤ 0.05 was considered statistically significant.

## Results

The study included 273 nurses from the Mansoura Oncology Center, with a mean fatigue score of 16.2 ± 7.5, and a median score of 16. The results revealed that 24.5% of nurses experienced grade IV fatigue (severe fatigue). Regarding work burden, the mean score was 7.7 ± 4.1, with a median of 7, and 68.1% of nurses reported grade D work burden, indicating high levels of work-related stress and strain (Table 1).

**Table 1:** The overall pattern of cumulative fatigue & work burden in 273 nurses

Fatigue grade	*N* (%)	Burden grade	*N* (%)
I	8 (2.9)	A	11 (4)
II	54 (19.8)	B	17 (6.2)
III	143 (52.4)	C	59 (21.8)
IV	68 (24.5)	D	186 (68.1)
Mean ± SD	16.2 ± 7.5	Mean ± SD	7.7 ± 4.1
Median (min-max)	16 (0–39)	Median (min-max)	7 (0–15)

### Independent predictors of fatigue and burden

Logistic regression analysis identified several factors associated with grade IV fatigue. Being married (AOR = 2.8, 95% CI: 1.5–5.2), holding a Bachelor’s degree or higher (AOR = 2.2, 95% CI: 1.1–4.4), and having less than 10 years of work experience (AOR = 2.4, 95% CI: 1.2–4.7) were found to be significant independent predictors of grade IV fatigue (Table 2).

**Table 2:** Association and predictors of fatigue (grade 4) among studied nurses

Variable	Total	Fatigue *N* (%)	Univariate analysis	Multivariate analysis
			COR (95%CI)	AOR (95%CI)
Overall	273	68 (24.9)		
Age:				
<30 years 30 years & more	152 121	47 (30.9) ** 21 (17.4)	2.1 (1.2–3.8) ** r	
Sex:				
Male Female	46 227	6 (13.0) 62 (27.3) *	r 2.5(1.01–6.2) *	
Residence:				
Urban Rural	185 89	44 (23.8) 24 (27.3)	r 1.2 (0.7–2.1)	
Marital status:				
Married Not married	156 117	49 (31.4) ** 19(16.2)	2.4 (1.3–4.3) * r	2.8 (1.5–5.2) *** r
Qualification:				
Nursing diploma Bachelor’s degree & above	100 173	15 (15.0) 53 (30.6) **	r 2.5 (1.3–4.7) **	r 2.2 (1.1–4.4) *
Monthly salary:				
Enough/enough & save Not enough	90 183	20 (22.2) 48 (26.2)	r 1.2 (0.7–2.3)	
Work duration:				
< 10 years 10 years and more	161 112	52 (32.3) *** 16 (14.3)	2.9 (1.5–5.3) *** r	2.4 (1.2–4.7) ** r
Department of work:				
In-patient Out-patient Operative room Intensive care unit	85 65 33 90	18 (21.2) 15 (23.1) 9 (27.3) 26 (28.9)	r 1.1 (0.5–2.4) 1.4 (0.6–3.5) 1.5 (0.8-3.0)	
Burden:				
A B C D	11 17 59 196	0 5 (29.4) 13 (22.0) 50 (26.9)	0 1.1 (0.4–3.4) 0.8 (0.4–1.5) r	

* Significant at *P* ≤ 0.05, ** significant at *P* ≤ 0.01, *** significant at *P* ≤ 0.001

For work burden, being unmarried (AOR = 2.2, 95% CI: 1.3–3.9) and reporting insufficient income (AOR = 2.8, 95% CI: 1.4–4.3) were identified as independent predictors of grade D burden, reflecting higher levels of work-related stress and strain (Table 3).

**Table 3:** Association and predictors of burden (grade D) among studied nurses

Variable	Total	Burden *N* (%)	Univariate analysis	Multivariate analysis
			COR (95%CI)	AOR (95%CI)
Overall	273	186 (68.1)		
Age:				
<30 years 30 years & more	152 121	106 (69.7) 80 (66.1)	1.2 (0.7-2.0) r	
Sex:				
Male Female	46 227	36 (78.3) 150 (66.1)	1.8 (0.9–3.9) r	
Residence:				
Urban Rural	185 89	121 (65.4) 65 (73.9)	r 1.5 (0.9–2.6)	
Marital status:				
Married Not married	156 117	94 (60.3) 92 (78.3)	r 2.4 (1.4–4.2) ***	r 2.2 (1.3–3.9) **
Qualification:				
Nursing diploma Bachelor degree & above	100 173	67 (67.0) 119 (68.8)	r 1.1 (0.6–1.8)	
Monthly salary:				
Enough/enough & save Not enough	90 183	48 (53.3) 138 (75.7)	r 2.7 (1.6–4.6) ***	r 2.8 (1.4–4.3) ***
Work duration:				
< 10 years 10 years and more	161 112	117 (72.7) 69 (61.6)	0.6 (0.4-1.0) r	
Department of work:				
Inpatient	85	65 (76.5)	r	
Out patient Operative room	65 33	37 (56.9) 20 (60)	0.4 (0.2–0.8) ** 0.5 (0.2–1.1)	
Intensive care unit	90	64 (71.1)	0.8 (0.4–1.5)	

* Significant at *P* ≤ 0.05, ** significant at *P* ≤ 0.01, *** significant at *P* ≤ 0.001

## Discussion

The findings of this study provide valuable insights into the prevalence and predictors of work-related fatigue and burden among oncology nurses in Egypt. This is particularly important given the increasing global recognition of oncology nursing as a high-risk specialty for occupational fatigue due to the emotional intensity and clinical demands involved [1, 11]. The results contribute to the growing body of evidence from low- and middle-income countries, highlighting the urgent need for targeted interventions to protect oncology nurses’ health and sustain quality cancer care services [23]. By comparing our findings with international studies and considering the specific Egyptian context, we can better understand the unique challenges and broader applicability of these results.

The overall rate of severe fatigue (Grade 4) among oncology nurses in the current study was 24.9%, which is lower than the 62% reported by Lee et al., 2022 in a Korean hospital setting during the COVID-19 pandemic [24]. This difference may reflect the extraordinary psychological burden associated with pandemic-related nursing care, where fear of infection and resource shortages significantly intensified fatigue [25]. Similarly, the lower prevalence compared to Polish nurses (35.3-50.5%) could be attributed to differences in measurement tools and healthcare system stressors [26].

Age was significantly associated with fatigue, with younger nurses (< 30 years) experiencing higher fatigue levels. These results align with findings from Korea by Jang et al., 2021 [27], suggesting that less professional experience and coping skills might contribute to vulnerability to fatigue. Tang et al., 2022 also observed that nurses aged 30–40 years with mid-level experience reported peak levels of cumulative fatigue, likely due to dual demands of clinical duties and emerging managerial responsibilities [19]. These patterns emphasize the importance of career-stage-specific interventions in managing fatigue among oncology nurses.

Gender differences were also evident, with female nurses nearly twice as likely to experience severe fatigue compared to their male counterparts. This finding is consistent with Tang et al., 2022 [19] and reflects ongoing gender role expectations, where women often face additional domestic burdens. However, as noted by recent studies [28, 29], gender gaps in occupational fatigue are narrowing in Western nations due to more equitable social and workplace policies. In Egypt, traditional gender roles remain influential, potentially exacerbating the fatigue experienced by female oncology nurses [30].

Marital status emerged as another predictor of fatigue, with married nurses experiencing higher fatigue rates (31.4%), corroborating findings from Iranian [31] and Korean studies [25]. These results suggest that family responsibilities might add to the occupational stress experienced by married nurses. In contrast, studies from Norway highlighted increased fatigue among single nurses, attributing it to social isolation and reduced emotional support [32]. This disparity may reflect cultural differences in family structures and support networks across countries.

Educational level was also associated with fatigue risk; nurses holding a bachelor’s degree or higher had greater odds of experiencing Grade IV fatigue (AOR = 2.2). This may be explained by the greater clinical and administrative responsibilities typically assigned to more highly educated nurses [33]. These findings are consistent with previous Chinese studies linking higher education with increased work expectations and fatigue [34].

Regarding work burden, the majority of participants (68.1%) reported Grade D burden, a rate substantially higher than the 27.2% reported by Tang et al., 2022 [19]. Predictors of Grade D burden in our study included being single and reporting insufficient income. Although Tang et al., 2022 observed slightly higher burden levels among married nurses, the association with low income was consistently reported [19]. This suggests that financial stress remains a critical factor in perceived work burden among oncology nurses, transcending cultural contexts.

Importantly, the high rates of fatigue and work burden identified in this study may have serious implications for patient care outcomes. Previous research has linked nurse fatigue with increased clinical errors, reduced empathy, and higher turnover rates, particularly in oncology settings where patient acuity is high [30, 35, 36].

The findings highlight an urgent need for targeted interventions to address work-related fatigue among oncology nurses. Nursing management should implement early warning systems to monitor fatigue levels and develop flexible scheduling to accommodate life stages and family responsibilities. Support programs focusing on female and mid-career nurses are critical. Financial incentives and mental health resources could further mitigate fatigue. Additionally, continuous professional development focused on resilience and workload management is essential. These strategies can improve nurse well-being, enhance patient care quality, and reduce turnover in oncology settings.

### Limitations

Several limitations should be considered when interpreting the findings of this study. First, the cross-sectional design restricts the ability to establish causal relationships between the identified factors and work-related fatigue and burden among oncology nurses. Second, data were self-reported, which may have introduced response bias, particularly social desirability bias despite measures to mitigate it, such as anonymous participation and standardized data collection procedures. Third, important work-related variables such as actual working hours, the frequency of night shifts, and specific unit assignments (pediatric versus adult oncology) were not objectively measured. Future studies should employ longitudinal designs and incorporate objective metrics, such as electronic timekeeping and fatigue biomarker assessments, to better capture work patterns and physiological fatigue levels. Additionally, while the study included nurses from both adult and pediatric oncology units, differences between these groups were not analyzed separately; future research should explore this aspect to understand setting-specific factors influencing fatigue.

## Conclusion

This study highlights the significant levels of fatigue and work burden experienced by oncology nurses in Egypt. Severe fatigue was found to be associated with marital status, higher educational attainment, and shorter work experience. Similarly, greater work burden was linked to being unmarried and facing financial difficulties. These findings illustrate the complex interplay between personal and occupational factors that contribute to nurses’ stress and exhaustion. There is a clear need for healthcare institutions to implement targeted interventions that support nurses both emotionally and professionally. Strategies such as workload adjustments, financial support, and structured wellness programs can play a vital role in improving nurses’ well-being. Future research should explore these areas through longitudinal studies to evaluate the impact of such interventions over time.

## Data Availability

No datasets were generated or analysed during the current study.
